# Perceptions of the Mamma Mia program, an internet-based prevention strategy for perinatal depression symptoms

**DOI:** 10.1371/journal.pmen.0000138

**Published:** 2025-04-03

**Authors:** Patricia A. Kinser, Sara Moyer, Heather A. Jones, Nancy Jallo, Ayomide Popoola, Leroy Thacker, Sally Russell, Ellen Solstad Olavesen, Thea Sundrehagen, Megan M. Hare, Bridget Xia, Susan Garthus-Niegel, Silje Haga, Filip Drozd

**Affiliations:** 1 Virginia Commonwealth University School of Nursing, Richmond, Virginia,; 2 Virginia Commonwealth University Department of Psychology, Richmond, Virginia,; 3 Children’s Hospital of Philadelphia Department of Child & Adolescent Psychiatry and Behavioral Services, Philadelphia, Pennsylvania,; 4 Virginia Commonwealth University, School of Population Health, Richmond, Virginia,; 5 Regional Centre for Child and Adolescent Mental Health, Eastern and Southern Norway, Oslo, Norway; 6 Department of Psychiatry and Behavioral Sciences, Tulane University School of Medicine, New Orleans, Louisiana,; 7 Institute and Policlinic of Occupational and Social Medicine, Faculty of Medicine of the Technische Universität Dresden, Dresden, Germany; 8 Institute for Systems Medicine (ISM), Faculty of Medicine, Medical School Hamburg MSH, Hamburg, Germany; 9 Department of Childhood and Families, Norwegian Institute of Public Health, Oslo, Norway; Instituto Federal do Maranhão: Instituto Federal de Educacao Ciencia e Tecnologia do Maranhão, BRAZIL

## Abstract

The Mamma Mia program is an internet-based intervention designed to prevent and/or intervene with perinatal mental health symptoms, such as depression symptoms. The purpose of this mixed-methods study, which occurred in a virtual setting, was to evaluate the perceived acceptability, obtrusiveness, and preliminary perceived usefulness of the Mamma Mia program by postpartum mothers who had access to the program through a larger parent study. Quantitative data on perceived obtrusiveness of the program (Unobtrusiveness Scale) were collected from 1772 pregnant women participating in a larger, three-arm randomized controlled parent study (clinicaltrials.gov: NCT04300894); qualitative data were obtained through semi-structured one-on-one interviews with a subset of these postpartum mothers (n = 55). Participants reported they found the program to be beneficial, emphasizing its guided content, focus on self-care, integration of mindfulness and educational components, and trustworthiness. Participants appreciated the structured nature of the program and expressed a high level of trust in the program, which was crucial for its perceived value and effectiveness. However, reported obtrusiveness of the intervention increased as participants progressed through the postpartum period, suggesting an evolving user experience with the program. Participants offered suggestions for improvement to the program, such as that the program should be app-based, the program should be available for partners to use, and that the program requires some updates to images and postpartum content. Overall, the study findings highlight the participants’ perceptions that the program is acceptable and useful. Future work is warranted to explore the enhancements proposed by participants to further optimize the program’s impact.

## Author summary

The Mamma Mia program is an internet-based intervention designed to prevent and/or intervene with perinatal mental health symptoms such as depression. In a study with a large number of pregnant women recruited through the United States who we followed throughout pregnancy and into the postpartum period, we evaluated usability, obtrusiveness, and areas for improvement of the Mamma Mia program. Participants found the program to be beneficial overall; they appreciated its guided content, focus on self-care, integration of mindfulness and educational components, trustworthiness, and activities such as breathing exercises and relaxation practices. Participants reported that usability was occasionally difficult in the postpartum period due to their time constraints as new parents. Participants offered suggestions for improvement to the program; for example, that the program should be app-based, the program should be available for partners to use, and that the program requires updates in images and postpartum content.

## Introduction

Perinatal mental health symptoms are a significant public health concern. In particular, symptoms of depression affect up to 20% of women during pregnancy and postpartum [1,2]. Depressive symptoms during the perinatal period can relate to a range of negative outcomes for the mother, including chronic mental health issues, impaired functioning, and reduced quality of life [3]. Untreated symptoms can affect the mother-infant bond, breastfeeding [3], the child’s social, emotional, and cognitive development [4], and increase the risk of mental health issues in partners [5]. They are also a significant societal burden due to consequent loss of quality of life, inability to work, the need for additional medical care, hospitalizations, and long-term treatment for both mothers and children [6]. Despite their high prevalence and impact, perinatal mental health symptoms such as depressive symptoms are often underdiagnosed and undertreated due to stigma, lack of awareness, and insufficient screening [7,8]. Addressing this gap is crucial for improving maternal and child health outcomes. Non-pharmacologic self-management strategies provide safe, accessible, and holistic options for managing mild and moderate perinatal depressive symptoms. Effective management and support can lead to improved outcomes and overall well-being for both mother and child, thereby lessening the burden on healthcare systems.

Digital interventions may deliver self-management strategies to a broad population at low cost and can mitigate barriers related to stigma, time, and logistics such as the need for childcare, thereby optimizing healthcare resource utilization. These interventions often include psychoeducation and self-help exercises and can be either unguided (i.e., self-help) or guided, facilitating easy access from home during pregnancy and the postpartum period. Several meta-analyses suggest that digital interventions for perinatal depression are effective [9–11], with guided interventions showing higher effect sizes than unguided [12], and can potentially even produce greater outcomes than in-person interventions [13]. In general, perinatal women report high acceptance and satisfaction with digital interventions, despite substantial heterogeneity in platforms, formats, and types of therapy [14,15]. However, digital interventions also face high attrition rates, ranging from 3.3% to 61.0% [15]. Although this can be mitigated to some degree by increasing guidance and reducing the number and length of sessions, understanding the acceptability and usefulness of digital interventions, alongside their effectiveness, is essential to address challenges to their use [16,17]. In the few acceptability studies on perinatal depression, internet-based cognitive-behavioral therapies are valued for their accessibility outside office hours and flexibility, making them less stigmatizing and scrutinizing compared to face-to-face therapy [18,19]. Even the more eclectic approach used in the Mamma Mia program was considered acceptable in a Norway-based study, due to its usefulness, ease of use, and credibility [20]. Mamma Mia is a universal preventive internet-delivered intervention offered to perinatal women (during pregnancy and postpartum), with the primary goals of preventing the onset or worsening of depression and enhancing subjective well-being during the perinatal period. In preliminary studies in Norway, the program’s strengths included its accessibility, allowing sessions at home or work, and its development by experts, which enhanced their trust in the program. It served as a “timeout” in the women’s busy lives and was appreciated for its accessibility, relevant information, increased awareness, normalization, and bonding with their baby [21], with 51% of women completing over 80% of the intervention in a randomized trial [22]. Despite its benefits, some women found the program stressful and time-consuming and desired more technical flexibility (i.e., being an app) and deeper coverage of topics such as infant sleep and multiparity [21]. This highlighted the need for further exploration of Mamma Mia’s acceptability and usefulness for perinatal depression in the United States (U.S.).

Assessing perceived acceptability and usefulness of this intervention is crucial for several reasons. Perceptions of acceptability and usefulness are strong motivators for engagement and sustained use [23]. If the intended user views the intervention as acceptable, they are more likely to engage with it leading to increased use and adherence over time, which is essential for the intervention to have its intended impact [24]. In fact, engagement has been demonstrated to be the most significant component of an intervention to improve health management and can be predictive of positive outcomes [16,25]. In addition, feedback from these perceptions assists researchers to identify any barriers or challenges encountered by participants that may negatively impact their acceptance and use. It is suggested that the evaluation of the usability by the person who will be using the intervention should be one of the first steps undertaken when assessing new digital interventions [26]. Failure to assess acceptability and usability may result in a digital intervention that is not used [26]. Thus, such information is incredibly useful for intervention improvement to maximize achievement of the intervention benefits and obtain positive outcomes.

The purpose of this mixed-methods study was to evaluate the perceived acceptability and preliminary perceived usefulness of the Mamma Mia program by pregnant and postpartum mothers in the U.S. who had access to the program through a larger parent study.

## Methodology

### Ethics statement

Ethics approval was obtained from the Virginia Commonwealth University Institutional Review Board (IRB #HM20017197) and informed consent was obtained from all participants via endorsement on electronic data capture system (REDCap).

### Study design and study participants

This study examined the perceived acceptability and usefulness, as part of a larger longitudinal mixed-methods parent study [27]. The parent study is a three-arm randomized controlled trial comparing 1) Mamma Mia (self-guided use of the internet-based program), 2) Mamma Mia Plus (self-guided use of the internet-based program plus occasional check-ins from research study staff to address barriers to use), or 3) Usual care (no access to the program, but same attention from study staff as the Mamma Mia group). The full study protocol is available on clinicaltrials.gov (NCT04300894), has been published previously [27], and is described in the Procedures section below. Briefly, the study setting was entirely virtual, allowing participants to engage from their preferred locations using their personal devices (e.g., smartphones, tablets, or computers). The parent randomized controlled trial enrolled 1953 pregnant women recruited nationally throughout the United States from October 2020 to July 2023 using national recruitment. The parent study participants met the inclusion criteria of being less than 25 weeks gestation at enrollment, age 18 and above, able to speak and understand English, have access to an internet-based program (e.g., via smartphone, tablet, or computer), and have a working phone number and email address.

The sequential mixed-methods study reported in this manuscript focuses on quantitative data from all three arms and qualitative data from a subset of the participants. The quantitative component includes n = 1,772 participants from the three arms who have completed baseline demographic data and study questionnaires about the program at 37-weeks gestation, 6 weeks postpartum, and 6 months postpartum. In order to best understand participants’ quantitative data about the obtrusiveness (or lack thereof) of the program, we subsequently analyzed qualitative data from interviews collected with *n* = 55 postpartum mothers who participated in the Mamma Mia or Mamma Mia Plus group and who volunteered for qualitative interviews upon completion of the program (approximately 6 months postpartum). Participants answered questions about their perceptions of the Mamma Mia program, the usefulness or lack thereof, and their suggestions for improvements. The parent study is still ongoing; therefore, not all parent study participants are included in this manuscript.

### Procedures

Parent study participants were recruited through national activities, such as social media, posts by social media influencers, and distribution of study information through perinatal-focused organizations; local targeted recruitment strategies complemented the national efforts, such as distribution of flyers at perinatal clinics, doula groups, clinician networks, and similar. It was indicated in the Mamma Mia program that it was developed by public health nurses and scientists/researchers. Individuals expressed interest in the study through a study-specific website. Study staff were available to answer any questions about the study. An electronic informed consent process was used (via an electronic data capture system, REDCap) in which interested individuals were provided with thorough information about the study purpose and procedures. Once an individual provided a digital signature in the electronic consent, a copy of the signed informed consent form was available to the participant. After an electronic informed consent process, enrolled participants completed baseline study questionnaires and were randomly assigned, using automated procedures via REDCap, to one of three study groups: Mamma Mia, Mamma Mia Plus, or usual care. Participants in the Mamma Mia arm received free access to the internet-based intervention. Participants in the Mamma Mia Plus arm received free access to the intervention, plus five direct phone contacts from study staff: twice in pregnancy, and three times during the postpartum period. Participants in the usual care arm engaged in their typical routines and prenatal and postpartum care and only engaged with the study at data collection timepoints. Continuous consent was obtained from participants throughout the study, at every timepoint for data collection. Continuous consent was obtained from participants at the start of the qualitative interviews, ensuring their continued interest in study participation.

### Intervention: The Mamma Mia program

First developed, tested, and refined in several studies in Norway [20,22,28,29], the Mamma Mia internet-based program was adapted for evaluation with a U.S.-based audience by this team of researchers in collaboration with community partners. Described thoroughly elsewhere [27,28], Mamma Mia involves 44 total modules designed to be initiated by the pregnant person prior to 25 weeks of pregnancy, with fully automated progression in the sequence of modules through 6 months postpartum. There are three phases of content delivery: the first phase- 11 sessions from the second trimester to the estimated due date; the second phase- 18 sessions in the first 6 weeks postpartum; and the third phase- 10 sessions from 6 weeks to 6 months postpartum. Users received email reminders during the day/week a module should be completed, in alignment with their respective stage of pregnancy or postpartum. The modules centered around the objectives of helping users cope adaptively with becoming a parent; engage in positive parent-infant interactions; engage in proactive and positive physical and mental health activities; seek help and support if mental health symptoms are present; and cope adaptively with mental health symptoms. The Mamma Mia program contains a variety of strategies to meet these objectives, such as normalizing psychoeducation about the perinatal period in an informal tone; brief cognitive and behavioral activities; frequent self-assessments of mental health symptoms; videos and audios to highlight infant behaviors and facilitate mindfulness practices; writing and reflective assignments; partner exercises, and similar. The program provides tailored feedback based on the mental health symptom self-assessments, such as providing suggestions for additional resources if there is a high depression score. Modular content is delivered in a “room” format, whereby there is a room focused on the pregnant/postpartum person, a room focused on the baby, and a room focused on connection with others [29]. We hypothesize that the Mamma Mia program may be helpful as a prevention intervention because it targets potentially mediating variables targeting emotional self-regulation, self-efficacy, and perceived social support [27,28]. The 44 modules contain educational information, evidence-based strategies and tools to encourage emotional self-regulation, and social support through computer-generated recommendations for engaging with one’s support network.

The Mamma Mia program is hosted by Changetech, a company that develops and facilitates internet- and mobile‐based lifestyle and behavioral change programs. At the time of this writing, the Mamma Mia program is only available to research participants.

### Data collection

Quantitative data collection consisted of questions administered via an electronic data capture system (REDCap) and qualitative data collection occurred via virtual semi-structured one-on-one interviews with participants.

#### Quantitative measures.

At baseline, participants self-reported demographic information, including age, race and ethnicity, education, employment, and household income. Perceived acceptability of the Mamma Mia program, defined as the match of the program with the participants’ daily life, was assessed at three timepoints (end of pregnancy, 6 weeks postpartum, and 6 months postpartum). This was captured using the four-item Unobtrusiveness Scale [30,31], which is assessed on a 7-point Likert-scale: 1) Using Mamma Mia fits with my everyday life; 2) Using Mamma Mia disrupts my daily routines (reverse scored); 3) Using Mamma Mia is practical for me; and, 4) Finding the time to use Mamma Mia is not a problem for me. Lower scores indicate obtrusiveness while higher scores indicate a lack of obtrusiveness (or unobtrusiveness). Unobtrusiveness was not collected at baseline, as participants did not have access to the program and would not be able to answer questions about its use. It was measured at all subsequent time points.

#### Qualitative interviews.

One-on-one semi-structured interviews were conducted with participants at approximately 4–6 months postpartum using Zoom-based technology. An interview guide was followed by interviewers with questions designed to assess participants’ experiences with the intervention and their perceptions of the acceptability and usefulness of Mamma Mia, with questions such as: “What strengths/benefits did you notice about Mamma Mia? What worked well for you? What weaknesses or disadvantages did you notice? Have you used any particular aspects of the program in your everyday life? What challenges do you identify might be present when encouraging others to use the program? Is there anything that could have been done to make it easier for you to use Mamma Mia?”

### Statistical analyses

#### Quantitative methods.

The JMP package was used for statistical analyses. Continuous variables were summarized using means and standard deviation while categorical variables were summarized using the frequency and percentage. Differences in continuous demographic variables were assessed using an equal-variance two-sample t-test while categorical variables were assessed using Fisher’s Exact test. A mixed linear model was fit for the obtrusiveness scale score (calculated as the sum of the 4 unobtrusiveness variables, possible range of 4 to 28) was fit. The model included fixed effects for Group (Mamma Mia vs Mamma Mia Plus), Time (37, 6 weeks postpartum, 3 months postpartum), and the interaction between Group and Time. This model also includes a random effect for subject to accommodate the within subject correlation. All analyses were done assuming an *α* = 0.05 significance level, unless otherwise specified, and conducted in R version 4.3.3.

#### Qualitative methods.

To further understand the quantitative findings about obtrusiveness of the program, we used a qualitative descriptive approach to explore participants’ perceptions of the acceptability and usefulness of the intervention. The analysis team (PK, NJ, SM) used the following steps. First, the team met to engage in reflexive discussion and address potential biases. Second, team members conducted an independent read of the transcripts line by line. Third, team members independently assigned codes to key concepts arising in the transcripts. Fourth, the analysis team came to a consensus on coding categories and identified initial themes. Fifth, the analysis team independently reviewed the transcripts to confirm/refute the draft themes. Finally, the team met again to finalize themes and identify quotes to illuminate key themes. To maintain the rigor and trustworthiness of the qualitative analysis, we employed common techniques as highlighted in previous research [32]. To enhance the validity of findings, all decisions were documented to maintain an audit trail [33]. To ensure the dependability and reliability of our analysis, we used peer debriefing in which the qualitative analysts discussed their analysis processes and the key findings with research team members who were not involved in the initial review [32].

## Results

Demographic information is presented in Table 1 below. As expected, there were no significant group differences with regards to demographics.

**Table 1:** Demographics.

Characteristic	Overall (n = 1772)	Control (n = 590)	Mamma-Mia (n = 592)	Mamma-Mia+ (n = 590)	*p*-value
Age, years (mean (SD))	30.70 (4.64)	31.10 (4.75)	30.38 (4.51)	30.63 (4.64)	.0783
Race/Ethnicity (n (%))					.8230
Asian/ Pacific Islander	88 (5.0)	27 (4.6)	27 (4.6)	34 (5.8)	
Native American/ Alaska Native	11 (0.6)	2 (0.3)	5 (0.8)	4 (0.7)	
African American/ Black	300 (17.0)	106 (18.0)	104 (17.6)	90 (15.3)	
Caucasian/ White	1167 (66.0)	387 (65.6)	381 (64.5)	399 (67.9)	
Latino/ Hispanic	149 (8.4)	49 (8.3	56 (9.5	44 (7.5)	
Other	54 (3.1)	19 (3.2)	18 (3.0)	17 (2.9)	
Education (n (%))					.2637
Elementary school	1 (0.1)	0 (0.0)	1 (0.2)	0 (0.0)	
High school	152 (8.6)	44 (7.5)	55 (9.3)	53 (0.0)	
Some college (at least 1 year)	213 (12.1)	70 (11.9)	71 (12.1)	72 (12.3)	
College degree	651 (36.9)	205 (34.7)	210 (35.7)	236 (40.2)	
Graduate degree	749 (42.4)	271 (45.9)	252 (42.8)	226 (38.5)	
Employed (n (%))					.4130
No	315 (17.9)	102 (17.3)	101 (17.3)	112 (19.1)	
Currently furloughed/unemployed	57 (3.2)	15 (2.5)	18 (3.1)	24 (4.1)	
Yes - hourly or prn	56 (3.2)	19 (3.2)	19 (3.2)	18 (3.1)	
Yes - part-time	230 (13.1)	74 (12.6)	91 (15.6)	65 (11.1)	
Yes - full-time	1102 (62.6)	379 (64.3)	356 (60.9)	367 (62.6)	
Income (n (%))					.7013
Less than $15,000	112 (6.4)	30 (5.2)	41 (7.0)	41 (7.0)	
$15,000-34,999	127 (7.3)	50 (8.6)	38 (6.5)	39 (6.7)	
$35,000-49,999	162 (9.3)	50 (8.6	60 (10.3	52 (8.9	
$50,000-99,999	474 (27.1)	161 (27.7)	156 (26.8)	157 (26.8)	
$100,000 or more	874 (50.0)	291 (50.0)	287 (49.3)	296 (50.6)	

### Obtrusiveness mixed linear model

**Fig 1:**
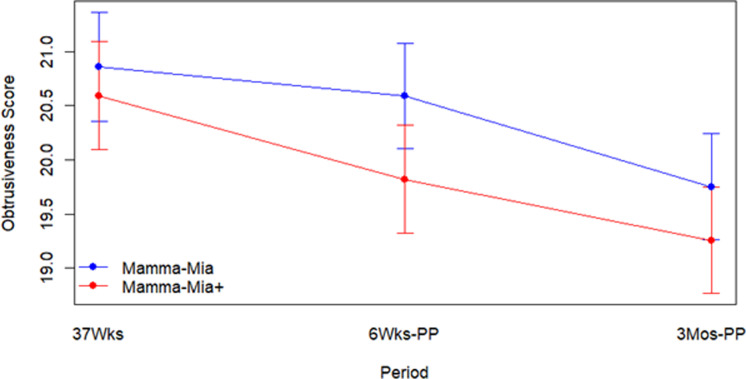
Obtrusiveness mixed linear model. Lower scores indicate more obtrusiveness on Unobtrusiveness Scale. Mamma Mia+ = Mamma Mia Plus; Wks = weeks; PP = postpartum; Mos = months.

When we fit the mixed linear model for obtrusiveness score, we found that there was a significant random effect for subject (χ²(1) = 855.755, *p* < .001). The unadjusted ICC was found to be 67.4%, while the adjusted r-squared for the model was found to be 68.2%. The Group by Time interaction term was found to be not statistically significant (F2,1368.53 = 1.24, *p* = .2895), so we refit the model without the Group by Time interaction and found that there was no significant Group difference (F1,927.19 = 2.81, *p* = .0938) but there was a significant Time effect (F2, 1370.51 = 28.88, *p* < .001), with lower obtrusiveness during pregnancy and higher obtrusiveness during the postpartum period. Graphical results for this model are presented in Fig 1.

Post-hoc tests indicated that there was a significant decrease in the obtrusiveness scores over time (indicating increased sense of obtrusiveness) with 37 weeks differing from both 6-weeks postpartum (Bonferroni adjusted p-value =.0040) and 3-months postpartum (Bonferroni adjusted p-value = <.001) and 6-weeks postpartum differing from 3-months postpartum (Bonferroni adjusted p-value <.001).

### Qualitative data

**Fig 2:**
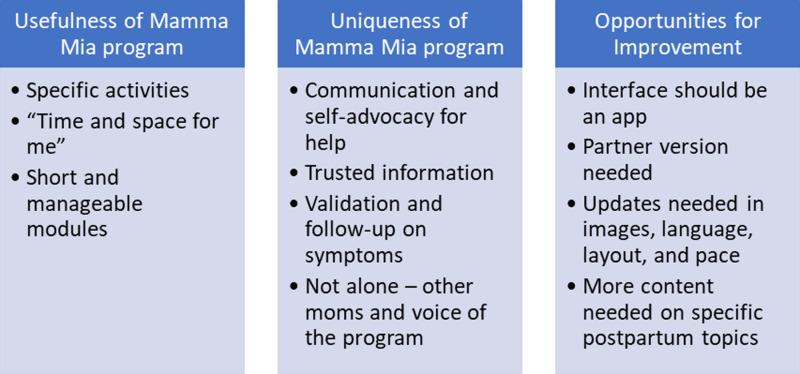
Summary of qualitative findings.

Fifty-five individuals participated in a postpartum interview about their perceptions of the intervention, with a focus on acceptability and usefulness (see Table 2 for self-described demographic data the qualitative participants). Representative quotes are provided to substantiate the key themes outlined below and in Fig 2.

**Table 2:** Demographics, qualitative participant subset.

Characteristic	Qualitative Participant Subset (n = 55)
Age, years (mean (SD))	30.09 (4.5)
Race/Ethnicity (n (%))	
Asian/ Pacific Islander	1 (1.8)
Native American/ Alaska Native	1 (1.8)
African American/ Black	9 (16.4)
Caucasian/ White	32 (58.2)
Latino/ Hispanic	10 (18.2)
Other	2 (3.6)
Number of children (n (%))	
One	33 (60)
More than one	22 (40)
Education (n (%))	
Elementary school	0 (0)
High school	3 (5.6)
Some college (at least 1 year)	9 (16.7)
College degree	19 (35.2)
Graduate degree	23 (42.6)
Partner status (n (%))	
Single	6 (10.9)
Living with partner	4 (7.3)
Married	44 (80)
Divorced/Separated	1 (1.8)

#### Theme 1: Usefulness of the Mamma Mia program.

Participants highlighted three key areas in which the Mamma Mia program was useful to them: 1) the specific guided activities; 2) the program encourages time and space for oneself; and 3) the modules were generally short and manageable.

##### Subtheme- specific guided activities:

 First, participants appreciated the specific guided activities in the Mamma Mia modules. Nearly half of the participants cited an appreciation for breathing practices, meditation/relaxation practices, and educational content about caring for themselves, their baby, and their relationships. Several participants highlighted that the variety of exercises and activities were helpful and that the program encouraged people to “build on the things worked on before” from week to week. Participants enjoyed the small snippets of advice and short videos demonstrating the content in the modules; with one participant noting this is “because reading something is different than seeing it. Actually being able to see examples, it helped a lot.” The two most commonly cited beneficial elements were with regard to mindfulness practices and learning to read infant cues. Multiple participants echoed the sentiment of a participant who said: “I liked learning how to read the [baby’s] cues…how to, for example, not run and pick up the baby as soon as he makes a little noise- that sometimes all he needs is to know that I’m sitting beside him, that I’m there.” Participants also appreciated the videos that demonstrated these concepts, saying that “[seeing] the mom with her baby- those are helpful just to be able to see it and not just reading about it” and that seeing “the different stages of the emotions for the babies and how to know the graduated comfort was a helpful tip.” Others ensured that their partners learned this same content with “videos of baby behavior” and written content about the stages of infant alertness: “that was super helpful, and I taught that to my husband.” Additionally, several participants found the breathing practices especially useful during stressful situations, like doctor’s visits, stating “I would listen to the recordings in the car on the way there and try to calm myself down.” A specific breathing exercise, “breathing up the spine,” was mentioned by one participant who said, “the breathing up the spine activity, I really liked that... I thought it was really useful.” As another participant suggested, many pregnancy-related apps do not have this kind of content: “I don’t feel like a lot of pregnancy things focus on [mindfulness] or have a way to walk you through it – it told you ‘stop, now we’re gonna do this breathing exercise’ so that was nice.”

##### Subtheme - “Time and space for me”:

Second, participants valued the program’s emphasis on self-care, with many expressing gratitude for being encouraged to take time for themselves. During pregnancy, participants expressed gratitude that the program encourages one to “appreciate the little moments” and “just having that time while I was pregnant to be more mindful about what I was going through has definitely helped me to slow down more and slow down, take things for what it is.” This concept was highlighted by one participant who discussed the relevance of this during the postpartum period, saying “It was a nice reminder to breathe, take some time for me- and the information was always very reassuring- a good reminder to myself to take a moment, even if it’s just to sit there and do nothing other than go through the lesson. Postpartum is really hard… so it was a nice reminder for me to go ‘okay, I can do this. This is normal.’” Many participants appreciated the reminders of “Hey, it’s ok to feel this way, and you might want to take some time for yourself.” Several participants reported that it was difficult to set aside time for the reflection and journaling aspect of the program, but they felt benefits when they did so: “the act of reflecting and journaling… it definitely helps when I’m able to take some time for myself and actually write out how I’m feeling, just being able to write something down definitely helps.” Another participant echoed this sentiment, saying the program reminded her she could “trust myself with the care of my body… the program went really well with that so I could check-in with myself.” A participant summarized her gratitude for the program in this way: “It was a great program, I’m really grateful for it… I think it made a big impact on my life in the last year.”

##### Subtheme - Short, manageable modules:

Third, the “short and manageable” nature of the Mamma Mia modules was highly appreciated by participants. Participants appreciated being able to focus on their mental wellness without having to schedule “a 45-minute session with a counselor where you have to find childcare or something.” The modules were described as “bite-sized” and “never felt like it was too much content in each session.” In general, despite some glitches with the functionality of the program described below in the third theme below, several participants reported that Mamma Mia was “pretty easy to navigate.”

#### Theme 2: Uniqueness of the Mamma Mia program.

Participants identified four key areas regarding the uniqueness of the Mamma Mia program: 1) the program emphasized learning about and supporting participant’s communication with others and self-advocacy for help; 2) participants felt they could trust the information in the program; 3) the program validated and followed-up on their symptoms; and, 4) participants felt “not alone” when using the program.

##### Subtheme - Communication and self-advocacy for help:

Of the four areas cited regarding the uniqueness of the Mamma Mia program, participants most commonly discussed that it was helpful for a perinatal program to emphasize communication techniques and self-advocacy. For many, this was seen as a significant difference from other perinatal apps that mainly focus on pregnancy stages or mindfulness. For example, several participants discussed learning skills to communicate their feelings with their partners: “I didn’t even realize some of the verbiage you could use [to talk about your feelings] before reading about it.” Another participant highlighted that “the most of the benefits [of the program] have actually been with the relationship aspect with my husband. A really hard piece is just trying to make sure… I’m not taking out my irritability or frustrations on him when they’re not because of him.” Many participants reported that they shared information learned from the program with their partners, such as about infant care. This was exemplified by a participant’s story of her excitement that modules would match her current experiences: “How to handle the crying baby and how to soothe and the overwhelm of breastfeeding... I felt like they were just so relevant to exactly where I was at the time. I would rush to my husband and be like, ‘Look at this. That’s exactly what we’re dealing with right now and we should try this.’ And it was really useful.” These sentiments were summarized by a participant’s comment: “The sections about communication were really good… because your communication just declines postpartum, you’re sleep deprived and depressed and stressed out and anxious. I thought that part was really good about communicating what you need support with… It was a good reminder for me to ask for help and not just try to do everything yourself.” Clearly, participants highly valued being encouraged to discuss their feelings with their partners.

##### Subtheme - Trusted information:

Second, participants appreciated the program’s trustworthiness, which was bolstered by its association with a respected research institution. This is coupled by knowing that Mamma Mia was developed by public health nurses and researchers, as highlighted in our recruitment process. One participant expressed confidence in the program’s information, saying, “With all the information out there- is it peer reviewed? I would trust an educational institution to vet certain resources more than I would myself.” This was echoed by another participant who stated, “It felt helpful to have outside advice coming from a place of research.” Participants appreciated the knowledge gained in the modules, as exemplified in one participant’s comment: “content-wise it was a very valuable program and I feel like it really helped me throughout the pregnancy and even birth… it approaches different topics that you don’t necessarily hear about or talk about in any of the [prenatal] classes.”

##### Subtheme - Validation and follow-up of symptoms:

Third, participants valued the program’s follow-ups on mental health symptoms, which encouraged them to be more honest about their feelings. One participant described the value of this as “knowing that I had the program to ask those questions and seeing them over and over and [knowing I should] answer them honestly as honestly as I can… I notice progress within myself each time I answer the questions, like I’m getting better each time. So, it helps me regulate my feelings and emotions I try to keep track of.” Another participant said that she received encouragement from the program to seek help for her symptoms because the program allowed her to “be more honest about [my symptoms]… I’m now in therapy because of some of the questions that were asked…I was like ‘maybe I do need help. I’m struggling a bit.’”

##### Subtheme - Not alone:

Finally, participants verbalized that the Mamma Mia program is unique because it helped them feel less alone in their experiences. Some participants described a sense of comfort knowing that other moms were using the program: “Considering that I know other women are doing it too, it definitely made me feel like I wasn’t alone during these stages.” This was particularly pertinent for participants who did not have a support system of other pregnant or parenting people around them, saying that Mamma Mia “was just a nice level of support… the only person who was having a baby was me. I didn’t really know anybody.” Another participant described how the impact of the images of women in the program shifted for her over time: “at first it was kind of funny, seeing the different women’s faces… but then by the end, I’m like, ‘These are my friends now’.” The program’s normalization of seeking help and its supportive nature were highly valued by participants, as summarized by one participant’s comment: “I got into the program and was reading about all these things and how it’s normal- I don’t need to go through this alone.”

#### Theme 3: Opportunities for improving the Mamma Mia program.

Although the feedback was often positive from participants about the program, almost every participant suggested opportunities for improvement in several areas such as: 1) the need for an app-based platform; 2) participants would like their partner to use the program; 3) the program requires image, language, layout, and pace updates; and, 4) there was a wish for more content in some areas.

##### Subtheme - Interface should be an app:

First, participants overwhelmingly suggested that the internet-based program should be in a mobile application (“app”) format. Almost every single participant cited frustrations with the current format, such as difficulties with trying “to log back on and go back through the program and see if I can look back at the stuff” and “having to open it through my email- some of the features didn’t work right.” Participants consistently expressed that the “interface could be nicer” and “an app would be easier.” There were also concerns about modules not matching their current pregnancy or postpartum state, such as “I had some issues with syncing up after I had given birth- it was asking me to do more things [as if I was] pregnant” – which participants felt could be addressed in an app-version. The current email reminder system was seen as clunky, and participants preferred push notifications from an app to complete modules. One participant noted, “If it was an app, I feel like it would be a lot easier. Something that can push a notification to your phone instead of an email, which can get easily lost.” Participants expressed a desire to be able to return to certain content or practices, which was difficult in the internet-based format: “Meditations or guided relaxation techniques, if there was a way to make those available outside of the lesson too, that might be helpful to go back to it and just by itself.”

##### Subtheme - Partner version:

Second, several participants expressed a wish for a partner version of the program. This was highlighted in a participant’s comment that “I wish there was a neutral one for parents; while I do think it’s helpful to have mom specific, I wish there was a postpartum one for dads- that could be really helpful.” Many participants described showing their phones to their partners when learning something new, but that it would be helpful for partners to have their own version to follow at their own pace. A new mother stated that Mamma Mia was helpful to see that her experiences were normal and it gave ideas for “ways to help cope- it really helped me out and it helped my husband out when I would show him and tell him, ‘Hey, this program is giving me advice and helping me this way. Maybe we can both try it and it’ll make parenting just a little easier for us.’” Participants described how there was key content regarding partner relationships which would have been helpful for both of them to have access to: “I read things and remembered ‘you two are in this together… remember you love each other and you’re not sleeping, it’s ok…. It’s not all about the baby’.” Participants suggested that, if a partner was doing their version, then the discussions about connecting between partners could be richer - “he didn’t know what I was necessarily going through, so it [could be] a nice reminder to touch base with him.”

##### Subtheme - Updates needed:

Third, some participants identified opportunities for improvement regarding updates to the images, wording, and layout of the program. Several participants felt that the wording needed more consistency across modules such that occasionally the verbiage would feel condescending, whereas it would feel nurturing in other areas. For example, one participant summarized this, saying “some of the content felt like I was being talked down to a little bit, and some of it felt very warm… it just felt inconsistent.” A few participants also felt that the “graphics felt a little weak sometimes… the pictures used felt a little dated and the facial expressions were kind of comical to me sometimes” particularly if the smiling faces did not match more difficult content being displayed. With regard to the layout or pacing, some participants made specific suggestions. For example, one suggested that progress bars in the modules could be helpful: “Design-wise, I found it frustrating that I didn’t know how long the module was going to be when I clicked on it… some took 2 minutes and some took 20 minutes. A progress bar would be really helpful.” Others suggested that the pace of modules was occasionally difficult, such as the increase in pace in the early postpartum “was hard to keep up… then all of a sudden I see an email that said I was behind.” Although recognizing that the content in postpartum is helpful, participants felt that it was “at times overwhelming, especially because fresh postpartum [I was] trying to take care of a baby.”

##### Subtheme - Wishes for additional content:

Finally, participants provided suggestions for additional content in some key areas centered around the postpartum period. One suggestion was to lengthen the availability of Mamma Mia up to “one year of parenthood, with check-ins as you go through different phases or stages with your child.” Another suggestion stemmed from a wish for discussions about infant feeding and related challenges. Several different mothers highlighted the need for support around the stress of infant feeding, such as one mother who stated, “a lot of the postpartum anxiety and depression that I suffered came from feeding worries.” Another suggested that her biggest stressor was about “pumping. That was probably the biggest stressors. Am I making enough milk? Am I going to keep making milk? Is this even going to work?” Other mothers suggested there was too much focus on breastfeeding but not enough regarding other options for infant feeding, for individuals who were unable to breastfeed. Other lesser-cited but relevant suggestions included more discussions regarding infant sleep, content about supporting siblings in welcoming a new baby, and a desire for more videos of seeing parents handle their infant behaviors.

## Discussion

Findings of this longitudinal mixed methods study indicated that the Mamma Mia program may be a useful and unique tool for depression symptom prevention during the perinatal period. Participants found the program to be unobtrusive during pregnancy but more difficult to keep up with over time during the postpartum period. Participants offered suggestions for improvement, highlighted in the discussion below.

### Usefulness of Mamma Mia

A primary theme that emerged from the study was that participants perceived Mamma Mia to be useful, driven by several distinct features. Participants valued the guided content, which encompassed active coping strategies like mindfulness, alongside educational resources preparing them for the significant life transition of parenthood. Research has consistently shown high acceptability of mindful activities among pregnant women [34]. Specifically, interventions incorporating mindfulness during pregnancy have been linked to enhanced childbirth-related self-efficacy efficacy [35], improved sleep quality [36], and better blood pressure management [37], contributing to overall wellbeing improvements.

The ease of manageability and conciseness of the Mamma Mia Intervention was also noted by participants, consistent with the literature on treatment acceptability of brief interventions in diverse pregnant people [38–40]. Additionally, pregnancy can be a period of heightened stress and decreased free time [41,42], making time-efficient interventions like Mamma Mia particularly appealing. Many mindfulness and other prenatal interventions can be time and labor intensive [34], which may affect the uptake of such interventions. Thus, a brief and easy to digest intervention like Mamma Mia allows users to work on managing their stress during pregnancy while also providing vital and interesting content.

### Uniqueness of Mamma Mia

The Mamma Mia program’s unique features were highly appreciated by participants, distinguishing it from other perinatal mental health interventions. The integration of mindfulness and active coping strategies within a structured, guided framework provided participants with practical tools to manage stress and prepare for the transition to parenthood. This approach not only addressed immediate mental health needs but also equipped participants with long-term skills for resilience. Furthermore, the program’s emphasis on self-care was noted as particularly beneficial, offering consistent reminders to prioritize personal well-being amidst the demands of pregnancy and early parenthood. The combination of educational content with practical, action-oriented steps made the program both informative and actionable. These unique aspects of Mamma Mia contributed to its perceived effectiveness and acceptability, setting it apart from more traditional, one-size-fits-all interventions. Future research should continue to explore these distinctive elements to further refine and enhance the program’s impact.

Participants expressed a strong sense of trust in the Mamma Mia program, which significantly contributed to its perceived value and effectiveness. This trust stemmed from the program’s development by public health nurses and researchers, ensuring reliable, evidence-based information. Trust is crucial for usability, encouraging consistent engagement and adherence [43,44]. For women from marginalized and minoritized communities, trust in medical providers can often be lower due to historical and ongoing healthcare disparities and structural racism in the healthcare system, which can undermine their confidence in medical advice [45,46]. Mamma Mia program may help bridge this gap, offering an accessible and reliable resource that participants feel comfortable using. Ensuring health interventions are perceived as trustworthy is essential to their success, particularly in diverse and underserved communities where trust in traditional healthcare systems may be less.

### Opportunities for improvement

Participants highlighted several areas for potential improvement in the Mamma Mia program such as in the program format, in availability for partners, and in postpartum period content gaps and obtrusiveness. First, regarding program format, many found the web interface challenging to navigate and suggested that transitioning to an app-based format would enhance usability. They emphasized the convenience of app-based interventions, noting their ability to push notifications directly to users—a feature particularly valued in the context of smartphone ubiquity [47] and proliferation of smart phone use (over 90% in the United States) [48]. Participants suggested that some images should be updated to help the program feel more fresh and modern. Participants expressed a wish for features such as a progress bar to improve their experience and pacing consistency. This suggestion is reasonable and warrants future work, given that developing a mobile app would enable easier navigation, the ability to push notifications, allow users to track progress seamlessly, and allow users to revisit content that they find helpful. Overall, these suggestions align with feedback from a qualitative study conducted on a Norwegian version of the Mamma Mia program [21].

Second, regarding availability for partners, several participants requested a partner version of the program. This suggestion is highly relevant given that partners often play a key role in family dynamics and maternal mental health, thus this could improve the overall family experience and enhance the program’s impact. Further, despite increasing recognition of maternal perinatal mental health, the mental wellbeing of partners during the transition to parenthood receives less attention from researchers and clinicians [49–51]. However, expectant partners are at heightened risk of depression and anxiety disorders, with perinatal depression affecting approximately 10% of fathers [52]. This not only impacts their own wellbeing but also influences family dynamics. For instance, there is evidence of a reciprocal relationship between paternal and maternal depression, highlighting the interconnectedness of parental mental health [53–56]. In addition, partner depression has been associated with an increased risk of behavioral and developmental problems in children, as well as poorer father-child relationships [ [58]. For those pregnant people with a partner, addressing the partner’s mental health is crucial as their involvement in family life grows, influencing maternal mental health, family cohesion, and child development [57]. However, societal norms around masculinity and stigma may discourage partners from seeking professional help, potentially leading to underreporting of their mental health challenges [50,58]. Furthermore, most studies on partners or co-parenting have traditionally focused on heteronormative relationships, examining the experiences of cisgender heterosexual couples, overlooking the diversity of modern families, including the multi-generational caregiving that is prominent in several racial and ethnic groups [59]. Involving any involved caregiver in the program could promote familial understanding and support. Broadening the focus from maternal to family mental health would represent a significant advancement in this field [60], aligning with current needs and contributing to a more comprehensive approach to perinatal mental health support. This is an important next step to advance the science in this field.

Third, regarding content gaps and obtrusiveness in the postpartum period, the need for more focus on infant feeding, sleep issues, and handling multiple children was frequently mentioned by participants. It is an important next step to evaluate whether and how to expand content offerings that address infant-related challenges, which could make the program more comprehensive for a wide range of postpartum scenarios. However, attention will need to be paid to whether it is feasible to increase content in the postpartum period, given that participants reported the program becomes increasingly obtrusive over time causing a decline in engagement. This is particularly concerning given the postpartum period’s heightened need for mental health support. As a result, future work is warranted to evaluate whether to simplify the program in the later stages, reducing the frequency of activities and focusing more on supporting users with fewer but high-impact resources.

### Limitations

The current study is not without its limitations. The subsample of participants interviewed in the qualitative portion of the study largely mirrored the diversity of the overall Mamma Mia sample, with a few exceptions, such as a higher proportion of married participants and those with higher levels of education. As a result, the generalizability of the study findings may be limited when applying them to non-married individuals and those with lower levels of formal education. Furthermore, the unobtrusiveness scale used was brief and may not have fully captured the range of perspectives on the acceptability of Mamma Mia as revealed in the qualitative interviews. Finally, the study did not account for the potential influence of participants’ previous internet use experience and how that may affect outcomes. Previous negative or positive internet use may influence engagement and adherence, as well as emotional responses [61]. To address this limitation, future research should consider assessing user experience at baseline and at scheduled times throughout a longitudinal study.

### Conclusions

The findings of this study underscore the acceptability of the Mamma Mia intervention throughout pregnancy in a U.S. population and emphasize the perspectives of a diverse participants in considering future improvements to the program. Serving as a mindfulness-based coping strategy supplemented with educational content, Mamma Mia proves to be a well-received, beneficial, and distinctive intervention. Future research should explore the practical implementation of the enhancements proposed by participants in this study to further optimize the program’s impact and reach, Table 3 highlights these proposed areas for improvement paired with action items for future consideration.

**Table 3:** Participants’ perceptions of opportunities for improvement and related actions.

Opportunities for Improvement	Action Item	Considerations and Potential Impact
Program Format	Development of a more user-friendly interface, e.g., a mobile app	This could address many issues highlighted by participants by enabling easier navigation, personalized push notifications, individualized content aligned with the user’s current perinatal stage, and progress tracking during each session. Additionally, an app interface could also allow users to revisit helpful content like mindfulness practices and relaxation exercises.
Content Gaps		
Participants desire a partner/support person version.	Develop a parallel program or log-in for partners/support people (offering them resources and guidance specific to their roles).	Including more interactive content between partners could improve partner communication and enhance the program’s effectiveness.
Participants request expanded content	Expand content available to make the program more comprehensive for a wide range of postpartum scenarios and experiences (e.g., dynamics of multiple children, different infant feeding journeys).	Addition of content in sessions for different parenting dynamics and postpartum experiences may offer more flexibility and allow more users to feel that the content is well aligned with their own experiences, potentially increasing effectiveness of the program.
Participants requested updates to graphics.	Incorporate graphics and content to ensure that the program resonates with a diverse range of users.	Addressing cultural and societal norms related to perinatal health, family structures, and mental health stigma is critical. The current format may not fully accommodate all user’s experiences, potentially making the program feel distant to some users.
Obtrusiveness and Usability		
As participants progress into the postpartum sessions, they report the program being increasingly obtrusive.	Simplify the program in the later stages, reducing the frequency of sessions.	Promote the user’s continued engagement with the program, possibly improving effectiveness.

Important next steps are to make the program more accessible through an app-based format; to evaluate whether partner/ support-person concurrent use could be helpful; and to evaluate ways to make the program less obtrusive during the postpartum period.
